# Exercise and Fat: A Primer

**DOI:** 10.1186/s40798-026-01085-y

**Published:** 2026-08-21

**Authors:** Alexander Braunsperger, Dominik Tischer, Ana Soriano-Arroquia, Martin Schönfelder, Anastasia Georgiadi, Henning Wackerhage

**Affiliations:** 1 Professorship of Exercise Biology, TUM School of Medicine and Health, Technical University of Munich, Munich, Germany; 2 Institute for Pharmacology and Toxicology, Biomedical Center, University of Bonn, Bonn, Germany; 3 Institute for Diabetes and Cancer, German Center for Diabetes Research (DZD), Helmholtz Munich, Neuherberg, Germany

**Keywords:** Exercise, Adipose tissue, Lipids, Fat metabolism, Fat oxidation, Thermogenesis

## Abstract

Over the past 25 years, adipose tissue has emerged as an organ comprising nearly 20 major cell types, including three types of adipocyte subtypes: white, brown, and beige. White adipocytes primarily store energy, brown adipocytes generate heat through thermogenesis and beige adipocytes exhibit intermediate characteristics. Distinct adipose tissue depots vary in their responses to external stimuli such as exercise, partly due to differences in developmental origin and progenitor cell populations. During exercise, adipose tissue contributes to whole-body energy metabolism by releasing lipids, which complement dietary fat intake as a major fuel source. Fat is the predominant substrate during moderate exercise intensities, and beyond its energetic role, lipid metabolism influences metabolic health through diverse signaling functions. Like other organs, white adipose tissue adapts to exercise training by undergoing structural and functional remodeling. Both endurance and resistance exercise stimulate the secretion of hormone-like signaling molecules, termed exerkines, derived from white adipose tissue (adipokines), brown adipose tissue (batokines) and skeletal muscle (myokines). These factors mediate exercise-induced adaptations in white adipose tissue to exercise stimuli and contribute to improvements in metabolic health. This narrative review covers: (1) fat as a chemical molecule in the context of exercise; (2) regulation of fat metabolism during exercise, including the relationship between exercise intensity and substrate oxidation; (3) adipose cell and tissue types; (4) adaptations of white and brown adipose tissue to exercise training; and (5) inter-organ crosstalk involving adipose tissue during exercise.

## Key Summary Points

- “Fat” comprises diverse lipid species and specialized adipocytes (white, brown, beige), which differ in cellular composition and play distinct roles in energy storage and expenditure.
- During exercise, triacylglycerols (fats) stored in adipose tissue and skeletal muscle lipid droplets are hydrolyzed to fatty acids that are transported to skeletal muscle and oxidized in mitochondria. Maximal fat oxidation rate per minute (≈ 0.4 g/min) occurs at moderate exercise intensity.
- Adipose tissue and skeletal muscle act as endocrine organs, release signaling molecules in response to exercise that promote adaptations such as browning of adipose tissue, increased lipolysis, vascularization and mitochondrial function.

## Introduction

The aim of this review is to broadly introduce sports and exercise scientists to fat metabolism and the function of white, beige and brown adipose tissue in the context of exercise. Many other reviews cover specific aspects of fat metabolism such as fat synthesis, termed lipogenesis [1], fat breakdown, termed lipolysis [2, 3], the function and regulation of fat metabolism during exercise [4, 5], different types of adipose tissue and their developmental origin [6], the cell types within adipose tissue [7], adipose tissues as endocrine organs that secrete adipokines and batokines [8, 9], heat generation by adipose tissue, termed thermogenesis [10], the pros and cons of high-fat diets in sport [11, 12], the effect of exercise training on fat loss [13], sex differences of adipose tissue and fat metabolism [14] and the relative energy deficiency in sport (RED-S) syndrome [15]. Note that the list of reviews is incomplete.

Key Points 

Fat is the body’s major energy reservoir and one of three macronutrients, alongside carbohydrates and proteins. “Fat” or “lipid”, a broader, more scientific term, is also used to describe several classes of metabolites that are non-polar and water insoluble.

Fat metabolism includes fat synthesis or lipogenesis and fat breakdown pathways such as lipolysis and β-oxidation. There are three types of fat cells or adipocytes and three tissues, termed white, brown and beige adipocytes and tissues.

There are many reasons why sports and exercise scientists are interested in fat. While fat is a major fuel source at rest and moderate but not intensive exercise and while specific fats and fat-soluble vitamins are essential for health, it is often viewed negatively. This is because excess body fat is associated with reduced performance, poor esthetics, and increased risk of metabolic and cardiovascular diseases. For instance, excessive fat lowers the power-to-weight ratio in endurance athletes, such as cyclists [16], and is often viewed unfavorably in sports like bodybuilding, where competitors aim for body fat levels below 10% during competition [17]. Beyond esthetics and performance, excessive adiposity is a risk factor for diseases such as type 2 diabetes and cardiovascular disorders, and exercise is one intervention to reduce fat mass and improve health in patients with metabolic disease. In contrast, low body fat levels in athletes due to prolonged low energy availability can lead to a syndrome known as RED-S [18–20]. Additionally, topics such as fat adaptation in endurance athletes [21], the concept of maximal fat oxidation (MFO) and Fat_max_ (i.e., the intensity where MFO occurs) [22], as well as hormonal and other regulation [23] of adipose tissue thermogenesis via molecules such as noradrenaline [24, 25] and irisin [26–31] have become areas of interest in sports and exercise science.

In this review, we first discuss fat as a chemical molecule, then fat metabolism and its regulation during acute exercise. We then review white, brown, and beige adipocytes and adipose tissues and how they adapt to exercise training. This is followed by a review of the communication in-between skeletal muscle and adipose tissue by hormone-like messengers termed myokines, adipokines and batokines.

### Fat as a Chemical Molecule: Fat Classes and Relevance for Exercise

“Fat” is a broad term whose meaning depends on whether it is used in the context of chemistry, nutrition, cell biology, or physiology. From a chemical viewpoint, fats or lipids are molecules that are non-polar and water insoluble. They can be classified into eight major categories each encompassing distinct subgroups: fatty acids, glycerolipids, glycerophospholipids, sphingolipids, sterol lipids, prenol lipids, saccharolipids and polyketides [32, 33]. The following six examples introduce different classes of lipids, their chemistry and give examples for exercise-related roles.

**Fig. 1:**
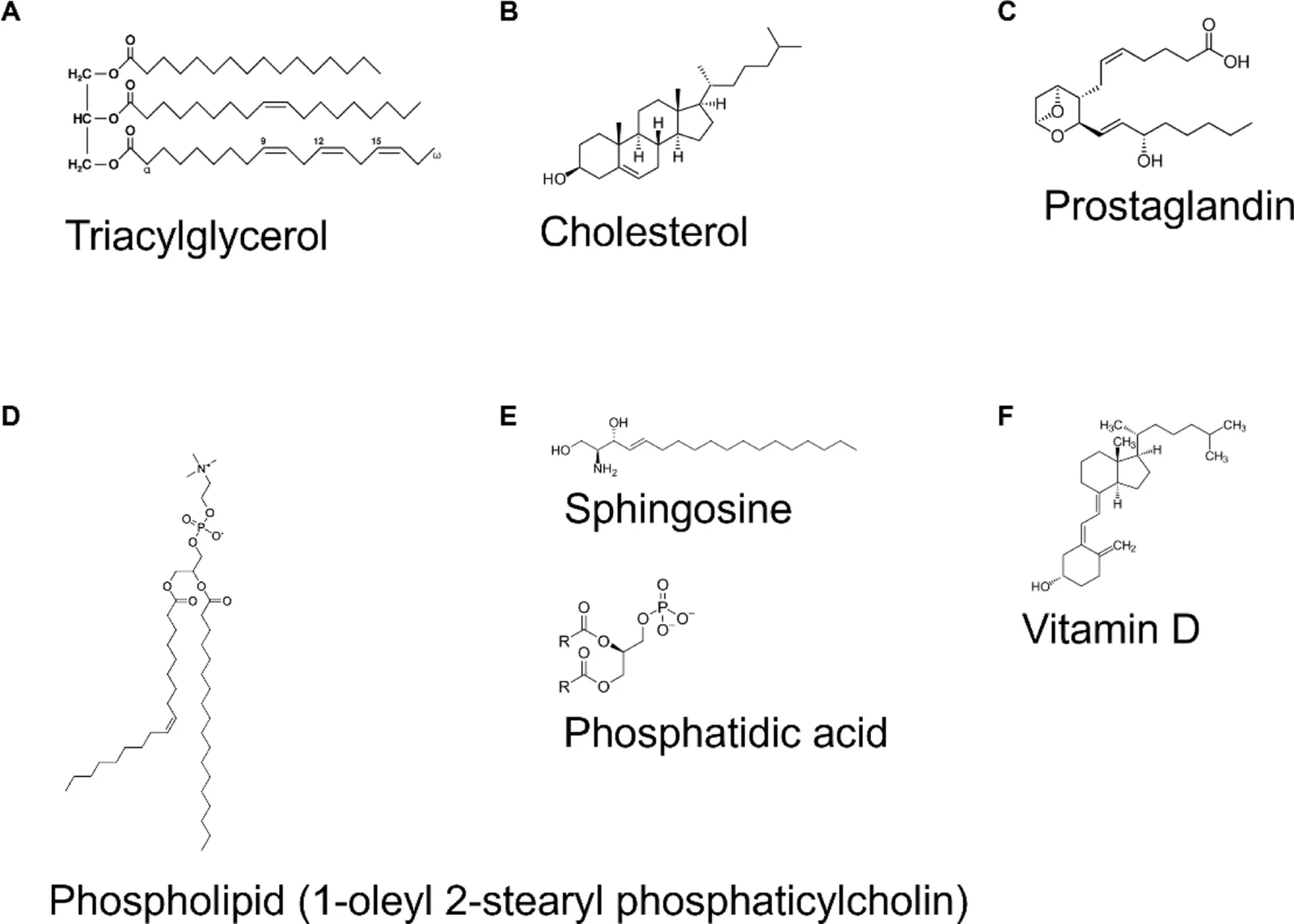
Examples of lipids in living organisms. **A** Triacylglycerol, **B** cholesterol, **C** prostaglandin, **D** phospholipid, **E** sphingosine and phosphatidic acid, **F** vitamin D are specific examples of lipids which play a role in the response to exercise

*Example 1. Triacylglycerol *(Fig. 1A) is the main storage fat and is also known as “triglyceride”. When we use the term “fat” we typically mean triacylglycerol. Triacylglycerols comprise a glycerol (i.e., a small metabolite with three alcohol groups (–OH)) esterified (i.e., bound to) with three fatty acids. Fatty acids themselves are carbon chains that are from two to over 22 carbons long. Furthermore, hydrogen atoms are attached to the carbons and there is a long carboxyl (–COOH) group at the end. Triacylglycerols provide together with carbohydrates the two main energy sources during exercise. Triacylglycerols contain approximately 9 kcal of energy per gram which is more than twice the energy density of carbohydrates and proteins that have an energy density of around 4 kcal per gram. During exercise, lipases such as adipose triglyceride lipase (ATGL) enzymatically hydrolyze each triglyceride molecule into one glycerol and three fatty acids [2, 34]. As a consequence, the levels of circulating glycerol and fatty acids increase during and after exercise as shown by metabolomics/lipidomics-based analyses [35].

*Example 2. Cholesterol *(Fig. 1B) is a lipid made from four fused carbon rings that is synthesized mainly in the liver or obtained through diet and circulates within low-density lipoproteins (LDLs) or high-density lipoproteins (HDLs) in the blood. LDLs with their lipid cargo can be taken up by cells via endocytosis, while HDLs facilitate cholesterol transport back to the liver and intestines for recycling or excretion [36]. Elevated LDL-cholesterol and low HDL-cholesterol concentrations are associated with an increased risk of atherosclerosis and cardiovascular disease [37, 38]. One mechanism by which exercise training improves health is through increases in HDL-cholesterol and decreases of LDL-cholesterol in response to both acute and chronic endurance exercise [39].

*Example 3. Prostaglandins *(Fig. 1C) are derived from 20-carbon fatty acids and have a cyclopentane (i.e., 5 carbon) ring. They are lipid mediators derived from polyunsaturated fatty acids, primarily arachidonic acid. Prostaglandins regulate body temperature, cardiovascular function and inflammation [40]. Athletes frequently use nonsteroidal anti-inflammatory drugs (NSAIDs), which inhibit cyclooxygenase activity and thereby reduce prostaglandin synthesis, to alleviate pain and inflammation associated with exercise [41].

*Example 4. Phospholipids* (Fig. 1D) are like triacylglycerols, but instead of having three fatty acids, the third position on the glycerol backbone is occupied by a phosphate group, usually linked to a polar head group. This structure gives phospholipids a hydrophilic head and two hydrophobic tails. This metabolite is both fat and water-loving which makes phospholipids the main component of cell membranes. Another role of exercise is that endurance training changes the phospholipid composition in skeletal muscle and this is linked to improved insulin sensitivity [42, 43].

*Example 5. Sphingosine *(Fig. 1E) is a lipid which is an 18-carbon molecule with an amino group (–NH2) and two alcohol groups (–OH). When it is phosphorylated, it becomes sphingosine-1-phosphate (S1P), a signaling molecule that can bind to S1P receptors. Sphingosine levels increase in plasma following high-intensity endurance exercise [44], and its oxidation contributes rough adaptation of skeletal muscle in response to exercise [45]. Another example of a signaling lipid is *phosphatidic acid* (Fig. 1E) which is a phospholipid whose concentration increases in mechanically loaded muscle. It activates mammalian target of rapamycin (mTOR) complex 1 signaling and protein synthesis and is discussed as a mediator of the muscle hypertrophy adaptation to resistance exercise [46, 47].

*Example 6. Vitamins A, D, E, and K * are classified as lipids that are sometimes supplemented by athletes. For example, vitamin D (Fig. 1F) has been extensively studied in relation to exercise-relevant outcomes, including muscle function and the prevention of sarcopenia [48–50]. Alongside energy availability and calcium intake, vitamin D is required for good bone health and injury prevention in athletes [51].

In summary, lipids are essential metabolites in sport because they provide high-density energy for exercise, form and remodel cell membranes, act as signaling molecules regulating inflammation and muscle adaptation, influence cardiovascular risk, and include fat-soluble vitamins critical for muscle and bone health.

### Fat Metabolism During Acute Exercise

Carbohydrates and fats are the two main energy sources during exercise in healthy, fed women and men. While carbohydrates provide a rapid but limited energy source, fats are an almost inexhaustible yet “slower” fuel. Specifically, glycolysis can generate up to approximately 9 mmol of ATP per minute per kilogram of dry muscle, whereas oxidative phosphorylation from fat metabolism produces only about up to 5 mmol of ATP per minute per kilogram of dry muscle [52, 53]. These are just approximate estimates as the “glycolyticness” and mitochondrial density of an individual will influence their ability to resynthesize ATP through fat or carbohydrate metabolism.

Fat mobilization during exercise begins with lipolysis, the breakdown of triacylglycerols into three fatty acids and one glycerol mainly in white adipose tissue, termed peripheral lipolysis. Lipolysis also occurs from intramuscular triacylglycerol (IMTG) stored as lipid droplets within muscle fibers [54]. Peripheral lipolysis is activated by the exercise-induced rise of the catecholamines noradrenaline and adrenaline as well as other factors and is inhibited by lactate [3]. The key lipase that removes the first two fatty acids from glycerol is hormone-sensitive lipase (HSL). However, mice that lack HSL can still carry out lipolysis, and this has led to the discovery of ATGL as another lipase. Finally, monoglyceride lipase (MGL) removes the final fatty acid from the glycerol [2]. The released free fatty acids and glycerol enter the bloodstream where their concentrations increase during acute exercise [35]. As fatty acids have limited solubility in water, in the circulation they mainly bind to the abundant carrier protein albumin [55].

For ATP production, fatty acids are taken up by skeletal muscle fibers via transporters such as fatty acid translocase (FAT/CD36), plasma membrane fatty acid-binding protein (FABPpm), and fatty acid transport proteins (FATPs) [5]. Inside the muscle fiber, they are activated by long-chain acyl-CoA synthetases (ACSLs) to form acyl-CoAs, which are then converted to acyl-carnitines by carnitine palmitoyltransferase I (CPT1) and transported into mitochondria via the carnitine-acylcarnitine translocase (CACT). After reconversion by CPT2, acyl-CoAs undergo mitochondrial β-oxidation which is oxidation at the β-carbon, the second carbon atom from the carboxyl (-COOH) end of the fatty acid. This generates acetyl-CoA for entry into the tricarboxylic acid (TCA) cycle [5]. During each turn of the TCA cycle, two carbons from the acetyl group of acetyl-CoA are bound to four-carbon oxaloacetate to form the six-carbon citrate. As the cycle proceeds, these two carbons are released as CO₂ returning the molecule to the four-carbon oxaloacetate.

The percentage of energy that comes from fat at rest or during exercise can be estimated by the respiratory exchange ratio (RER). A RER of 0.7 is 100% fat oxidation and an RER of 1.0 is 0% fat oxidation. The absolute amount of fat oxidation in grams per minute can be estimated from equations such as Frayn’s Equation [56, 57] which calculates fat oxidation from oxygen uptake (V̇O_2_), carbon dioxide production (V̇CO_2_) and urinary nitrogen excretion:

$$Fat \, oxidation\left( {g/min} \right) \, = \, 1.67 \times VO_{2} {-}1.67 \times VCO_{2} {-}1.92 \times n.$$

A common approach is to assume a fixed value for urinary nitrogen excretion. Because urinary nitrogen excretion usually is not measured, a fixed value of 0.01 g/min⁻ can be assumed, consistent with commonly used indirect-calorimetry calculations by based on Frayn’s stoichiometric Equation [56]. This value is also in agreement with the 24-h urinary nitrogen excretion rates reported by Clauss et al. following 5 h of exercise [58]. Thus, fat oxidation can be estimated just from V̇O_2_ and V̇CO_2_ data. With increasing exercise intensity, overall energy expenditure increases linearly with oxygen uptake while the fat oxidation tends to decrease. As a consequence, absolute fat oxidation increases with rising exercise intensity and reaches MFO or Fat_max_ at medium intensities of 50–65% of the V̇O_2_max [59] before declining and approaching zero from about 85% of the V̇O_2_max [60, 61]. This shift from predominantly fat oxidation to predominant carbohydrate usage with increasing exercise intensity has been termed the “crossover” concept by Brooks and Mercier [62].

A key question is the mechanism underlying the “crossover” concept. In their review, Lundsgaard et al. conclude that the rate of glycolysis is the primary determinant of the amount of fat oxidized during exercise [5]. Remarkably, this is the same assumption made by Alois Mader in his mathematical model of human energy metabolism [63, 64]. Specifically, at low exercise intensities, the glycolytic production of pyruvate and thus acetyl-CoA is insufficient to meet the mitochondrial demand for acetyl-CoA, so the assumption is that the “missing” acetyl-CoA is supplied by β-oxidation of fatty acids. As exercise intensity increases, glycolytic flux rises more than the mitochondrial demand for acetyl-CoA, progressively closing the gap between glycolytic acetyl-CoA supply and mitochondrial acetyl-CoA demand, until glycolysis alone can meet this requirement. In this model, acetyl-CoA from β-oxidation is simply a gap filler, providing the acetyl-CoA that is lacking at lower exercise intensities where glycolytic flux is too low to meet the mitochondrial acetyl-CoA demand.

The hypothesis that “glycolytic flux determines the rate of fat oxidation” is also consistent with the observation that fat oxidation increases during prolonged exercise and in other situations that induce glycogen depletion [65]. Mechanistically, low glycogen availability reduces glycolytic flux [66, 67], thereby decreasing the mitochondrial supply of glycolysis-derived pyruvate and acetyl-CoA. To maintain acetyl-CoA provision for the TCA cycle at a given power output, β-oxidation increases as a compensatory mechanism, resulting in a higher rate of fat oxidation.

**Fig. 2:**
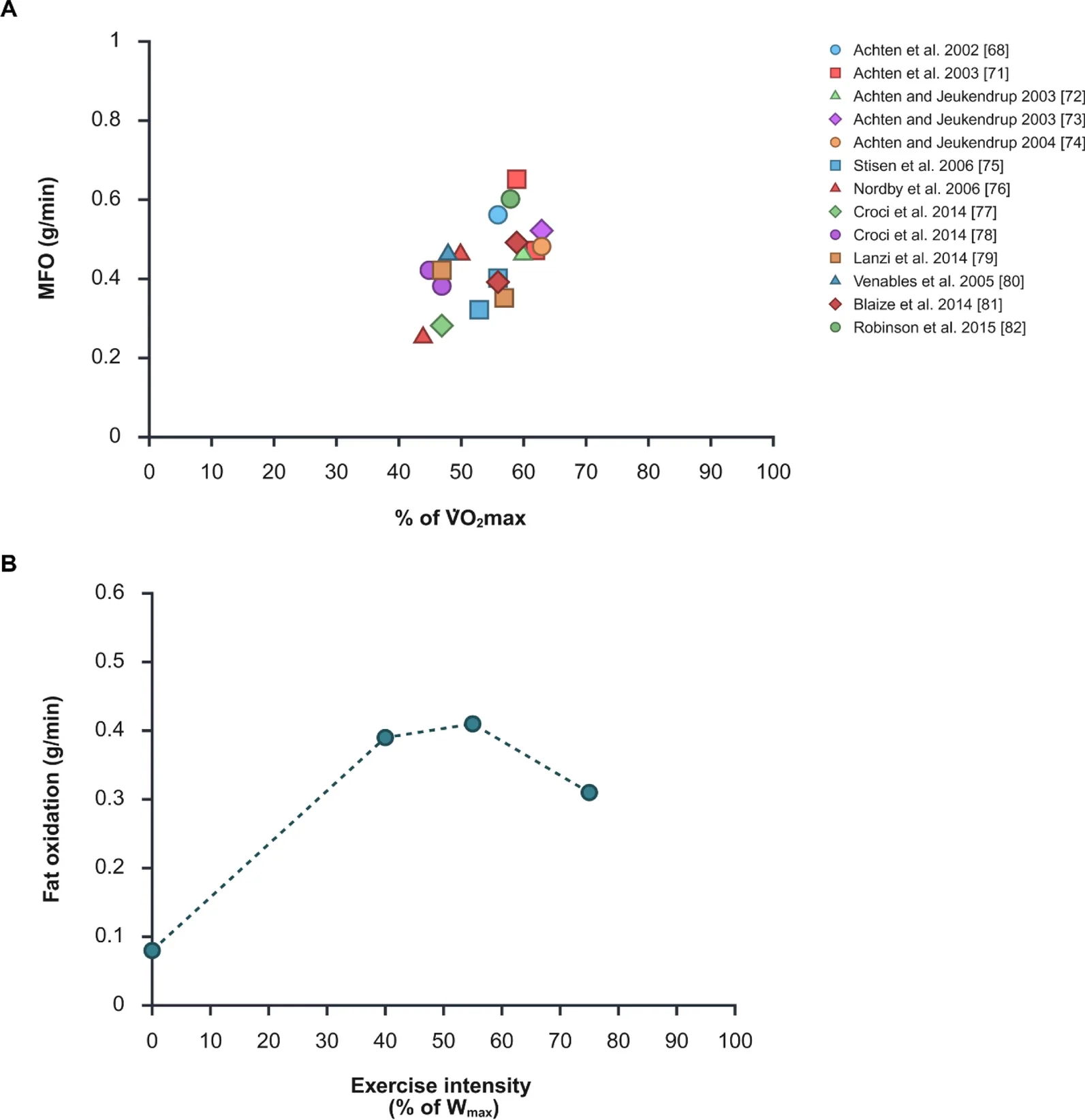
**A** Descriptive overview of previously reported maximal fat oxidation (MFO) values in relation to exercise intensity (% of V̇O₂max) of previously published studies as compiled in Randell et al. [71]; no pooled estimates calculated. Relationship of individual MFO and relative exercise intensity (% of V̇O_2_max) among athletes occurs at 50 ± 15% of V̇O_2_max [71]. Data are derived from multiple studies [69, 72–83]. **B** Example of the within-study relationship between exercise intensity and fat oxidation during cycling at 40% (0.39 ± 0.04 g/min), 55% (0.41 ± 0.04 g/min) and 75% (0.31 ± 0.02 g/min) of W_max_ from data as compiled in Loon et al. [61]. Relationship of fat oxidation (g/min) and exercise intensity (% of W_max_) was assessed during exercise in trained male cyclists. Mean values were plotted in the graphs

The maximal rate of fat oxidation and Fat_max_ are determined using an incremental step test with spirometry up to an intensity where the RER exceeds 1.0 [68, 69]. Generally, in steady-state conditions, RER values of above 1.0 are usually interpreted as de novo lipogenesis. However, during exercise values of RER > 1.0 depend on the disequilibrium of the body’s bicarbonate pool since the body buffers the production of lactic acid by using bicarbonate. This reaction generates additional CO_2_ that does not derive from substrate oxidation [70]. The maximal rate of fat oxidation and Fat_max_ values depend on nutritional status as fasting, fat-rich diets and glycogen depletion increase the maximal rate of fat oxidation and change the Fat_max_ intensity. The maximal rate of fat oxidation values also depends on endurance training status as trained athletes have a higher V̇O_2_ at the Fat_max_ intensity where the maximal rate of fat oxidation is reached than untrained persons. In 933 male and 188 female competitive athletes of different disciplines, the maximal rate of fat oxidation was on average 0.59 ± 0.18 g/min (range: 0.17–1.27 g/min) occurring on average at a Fat_max_ of 50 ± 15% of the V̇O_2_max (range 22.6–88.8%) [71] (Fig. 2A).

At the extreme end, male ultra-endurance runners on a low-carbohydrate diet can reach MFO values of 1.54 ± 0.18 g/min [84]. Notably, such rises in the MFO occur within as little as five days on a low-carbohydrate, high-fat diet [84]. While increased fat oxidation protects limited glycogen stores, there is a physiological cost. Fat oxidation results in a higher oxygen demand during exercise which means that the athlete will need ≈10% more oxygen to resynthesize 1 mol of ATP [85, 86]. The better oxygen-efficiency of carbohydrates probably explains why Sherpas have a poorer capacity for fat oxidation and a better capacity for carbohydrate usage when compared to lowlanders [87]. Importantly, while exercise training increases fat oxidation at the same absolute workload, it does not significantly alter 24-h fat oxidation rates [88]. When fat oxidation was assessed during exercise, intensities around 50% of maximal power output (W_max_) during cycling showed highest substrate utilization of fats. Fat oxidation was measured at 0.08 ± 0.01 g/min at rest, 0.39 ± 0.04 g/min at 40%, 0.41 ± 0.04 g/min at 55% and 0.31 ± 0.02 g/min at 75% of W_max_ (Fig. 2B) [61]. The maximum fat oxidation and Fat_max_ are also investigated in the context of health. A meta-analysis has concluded that training at the Fat_max_ intensity reduces body weight and body fat in people with obesity [89], but it is not clear whether training at Fat_max_ intensity is more effective than comparable training at other intensities.

### White, Brown, and Beige Adipocytes and Adipose Tissue

Historically, adipose tissue was often regarded as an inert, unhealthy, lipid-containing component of the body, overshadowed by skeletal muscle in exercise research. However, the obesity pandemic, the discovery that adipose tissue acts as an endocrine organ secreting adipokines such as leptin [90, 91], and advances in the anatomical and functional characterization of adipose depots [6, 92] have turned fat or adipose tissue into an interesting tissue for sports and exercise scientists. There are several adipose depots throughout the human body and each depot originates from different progenitor cells, which results in characteristic functions of each fat pad, e.g., energy storing white adipose tissue and heat-generating brown adipose tissue [93]. The amount and type of adipose tissue also play a role in sports. For example, gymnasts, runners, and bodybuilders all aim to limit body fat levels leading up to their competitions [17, 94–96]. In contrast, sumo wrestlers aim for body fat percentages above 25% [97], while﻿ cold water swimmers rely on more active brown adipose tissue to generate the necessary heat for maintaining a healthy core body temperature [98]. This section introduces white, brown and beige adipose tissue, their anatomical distribution, endocrine functions, and the role of brown adipose tissue in thermogenesis.

### White Adipocytes and White Adipose Tissue

**Fig. 3:**
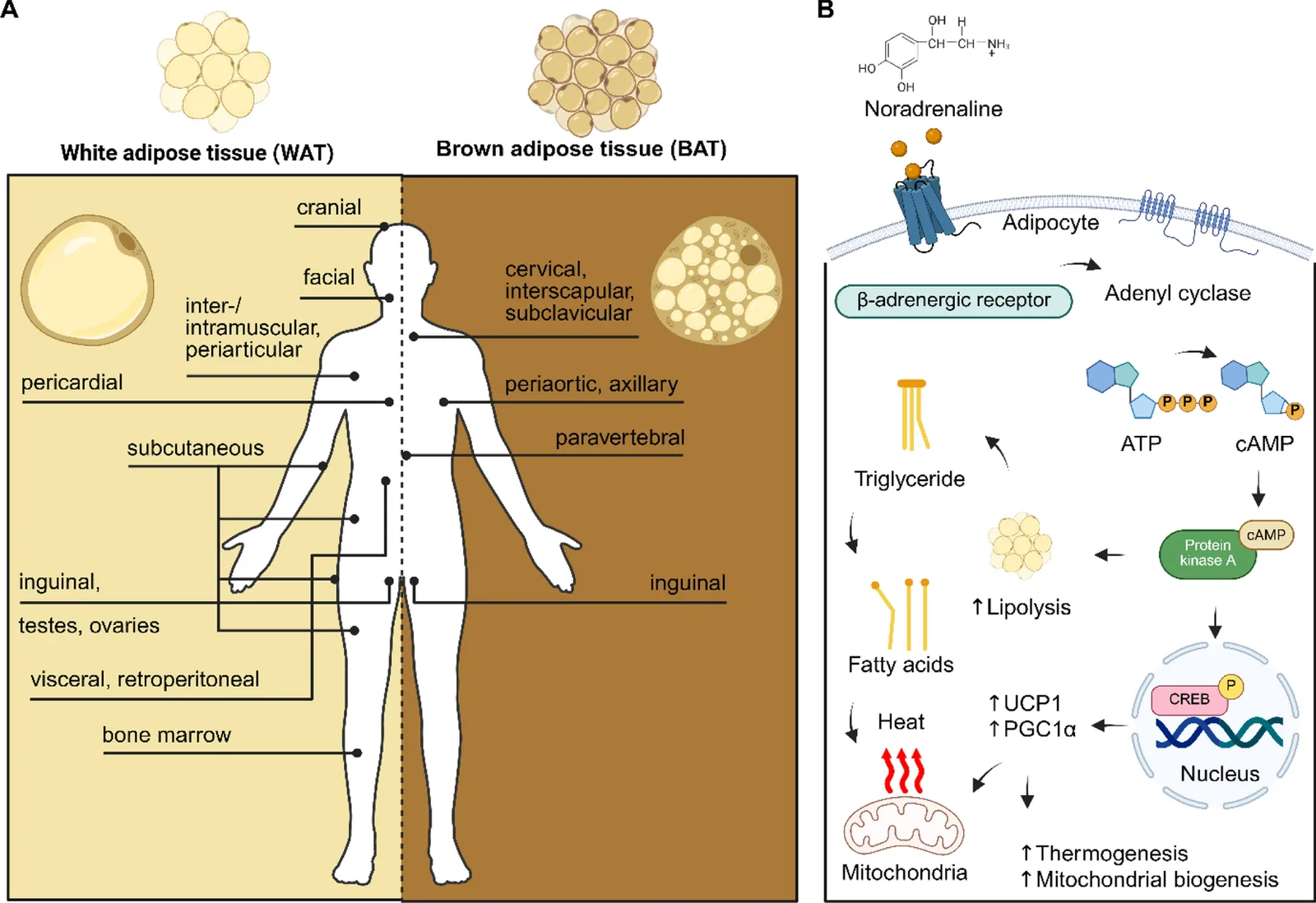
**A** Distribution of major human white and brown adipose tissue depots. **B** Schematic overview of noradrenaline-mediated thermogenic signaling in adipocytes. Original figure created by the authors. *WAT *white adipose tissue, *BAT *brown adipose tissue, *ATP *adenosine triphosphate, *cAMP *cyclic adenosine monophosphate, *CREB *C-responsive element-binding protein, *P *phosphate, *UCP1 *uncoupling protein 1, *PGC1α *peroxisome proliferator-activated receptor gamma coactivator 1 alpha

Body fat or adipose tissue can range from 5% of body weight in very lean individuals to over 60% of body weight in severely obese individuals [99]. Body fat is mainly stored as WAT, distributed throughout the human body (Fig. 3A) with functions extending beyond energy storage. WAT not only comprises white fat cells, termed “white adipocytes” but nearly 20 different types of cells including vascular, immune, and progenitor cells [7, 100–102]. Single-cell sequencing has revealed differences in the cell composition of different depots. Within the depots, some cell types are associated with metabolic health or disease risk such as human-specific subcutaneous adipocyte subtypes (called h-Ad-7) [100], metallothionein-expressing CD8-positive T cells [103] (type 2 diabetes) and type 1 innate lymphoid cells that are associated with low-grade inflammation [104]. White adipocytes have a single large lipid droplet and relatively few mitochondria. WAT depots are found under the skin, termed “subcutaneous”, and around the internal organs, termed “visceral”. Visceral fat can further be distinguished into fat pads in the groin area or around the reproductive organs, abdominal fat pads between the intestines (mesenteric) or on the stomach (omental) and finally a fat pad on the heart sac (pericardial) (Fig. 3A). The deposition of adipose tissue within subcutaneous WAT depots is associated with depot-specific roles. For example, subcutaneous WAT around the hips, buttocks, and thighs, termed “gluteofemoral depot”, is associated with good cardiometabolic health, whereas increased visceral fat is associated with a higher risk of cardiovascular disease and metabolic disorders [105]. Moreover, the gluteofemoral depot secretes different adipokines when compared to abdominal adipose tissue such as adiponectin and the insulin-sensitizing lipokine palmitoleate [106, 107]. Additional fat is in the bone marrow and skeletal muscle [108, 109].

### Skeletal Muscle Fat Depots

Skeletal muscle fat includes intramyocellular (intramuscular) lipid droplets, abbreviated as IMCL, and intermuscular adipose tissue, abbreviated as IMAT. Both IMCL and IMAT affect muscle function, metabolism, and can contribute to disease. How IMCL and IMAT levels affect health is complicated because both endurance athletes with high insulin sensitivity and insulin-resistant individuals have high IMCL content, which is known as the athlete’s paradox. Also, aging and diabetes are associated with intramuscular fat deposition, which impairs insulin signaling and muscle performance [110–112]. Early work by van Loon and Goodpaster (2006) proposed that the balance between IMCL content and muscle oxidative capacity explains the athlete’s paradox, a concept refined by recent advances in lipidomics and skeletal muscle imaging [113]. Transmission electron microscopy analysis of skeletal muscle biopsies from endurance-trained athletes revealed 24% greater total contact length between lipid droplets and mitochondria compared to untrained controls [114]. Consistent with this view, endurance-trained men have a greater lipid-stained muscle area than men with obesity or type 2 diabetes despite superior insulin sensitivity [115]. The IMCL of stained muscle sections from male athletes showed a significantly higher lipid area (4.3 ± 0.5%) than from obese (2.2 ± 0.5%) or diabetic (2.3 ± 0.4%) men [115]. Moreover, endurance exercise training in insulin-resistant individuals increases intramuscular triacylglycerol content while reducing bioactive lipid intermediates such as diacylglycerols and ceramides, coinciding with improved insulin sensitivity [116, 117]. Ceramides appear strongly linked to insulin resistance and in contrast diacylglycerols can be elevated in endurance-trained muscle without metabolic impairment [118]. However, the relative contributions of ceramides versus diacylglycerols to insulin resistance are still discussed in the literature [116, 118–120]. Subsequent studies have demonstrated that lipid intermediates such as diacylglycerols and ceramides are not uniformly deleterious, but depend on their molecular species and intracellular localization [121–123] as well as skeletal muscle mitochondrial oxidative capacity and lipid turnover [124].

Exercise also remodels lipid droplet morphology and distribution. Whereas individuals with type 2 diabetes accumulate large lipid droplets in glycolytic type II fibers, endurance-trained athletes store a higher number of smaller lipid droplets in oxidative type I fibers, closely associated with mitochondria [125]. Exercise training can induce this athlete-like phenotype in previously sedentary individuals [125]. Collectively, these findings demonstrate that insulin sensitivity is determined by lipid species composition, droplet size and localization, fiber-type specificity and mitochondrial coupling rather than IMCL content per se, reframing the athlete’s paradox as an adaptive feature of trained skeletal muscle.

While exercise-induced IMCL accumulation represents a reversible and metabolically adaptive phenotype of trained skeletal muscle, fat infiltration under pathological conditions appears to arise from distinct cellular mechanisms. Recent single-cell RNA sequencing (scRNA-seq) studies have identified heterogeneous adipocyte subtypes within skeletal muscle fat depots, highlighting previously unrecognized regulatory roles of these cells in muscle metabolism and regeneration following injury [126, 127]. Particularly, fibro-adipogenic progenitors (FAPs) have emerged as a key cellular source of ectopic fat accumulation under pathological conditions. Dysregulated differentiation of FAPs, as observed in rotator cuff muscle degeneration, contributes to fatty infiltration within skeletal muscle, thereby impairing muscle repair, remodeling and functional recovery [128–131].

### Brown Adipocytes and Brown Adipose Tissue

Unlike WAT, BAT can generate heat to maintain the body’s core temperature. The heat-generating process is also termed “thermogenesis”. Like WAT, BAT does not only comprise adipocytes. Single-cell/nucleus analyses show that BAT comprises different types of brown adipocytes, adipocyte progenitors, vascular cells, dendritic cells and immune cells [102]. In contrast to WAT, BAT is rich in mitochondria and contains multiple smaller lipid droplets [6]. Its heat-generating or “thermogenic” function is mainly but not only due to uncoupling protein 1 (UCP1) that generates heat by uncoupling mitochondrial respiration from ATP resynthesis (Fig. 3B). Brown adipocyte thermogenesis is activated by the catecholamines adrenaline and noradrenaline as well as many other factors [24, 132]. Human BAT is located in the neck, near the clavicula, termed “supraclavicular”, around the heart sac, termed “pericardial”, and the vertebral column, termed “paravertebral” [109] (Fig. 3A). For unknown reasons, BAT activity declines with age while leaner individuals have more BAT activity than obese individuals. More specifically, random ^18^FDG PET/CT scans throughout a calendar year show that 20-year-olds have more active BAT (≈ 60%) than 60-year-olds (≈ 10%) [133]. Because BAT activation increases energy expenditure and glucose disposal it has led to research to target BAT thermogenesis to treat obesity and metabolic disease [134]. Individual BAT volume is associated with longevity and lower incidence of metabolic or cardiovascular disease [133]. Activated BAT takes up glucose and increases energy expenditure [135]. However, the in vivo quantification of BAT’s contribution to whole-body thermogenesis remains a challenge since ^18^FDG PET/CT scans rely on a radioactive tracer and might underestimate the total volume of activated BAT in response to cold exposure [136]. Lean individuals have a greater BAT volume, averaging 334 ± 188 ml compared with 130 ± 141 ml in obese individuals, and a higher prevalence of metabolically active BAT, with 38 ± 22% versus 7 ± 7%, respectively [135]. This association suggests that active BAT might contribute to metabolic health. One unexplained paradox is that exercise increases known BAT activators such as noradrenaline [137] without seeming to increase BAT activity in humans following exercise training [138].

### Beige Adipocytes and Beige Adipose Tissue

Adipose tissue is, like skeletal muscle, a plastic tissue that can adapt to stimuli such as exercise or cold. A key example of this is that cold exposure leads to the emergence of “beige” or “brite” adipocytes in WAT that express UCP1 [139]. Such beige adipocytes are adipocytes distinct from white and brown adipocytes [140]. Beige adipocytes differentiate within WAT and therefore derive from different progenitor cells than brown adipocytes [6]. Beige adipocytes exhibit variable lipid droplet composition and mitochondrial density [141] and can transition between white- and brown-like states [92]. Under specific stimuli, such as cold exposure or β-adrenergic activation, beige adipocytes express more UCP1 protein and thereby increase their capacity for thermogenesis, a process termed “beiging” or “browning” [92]. Recent studies have identified a thermogenic subpopulation of white adipocytes (termed P2 in mice and h-Ad-3 in humans) utilizing ATP-dependent futile cycles, which contribute to total energy expenditure and are associated with improved metabolic health [142]. Additionally, a subset of beige adipocytes with high levels of tissue non-specific alkaline phosphatase (TNAP) utilizes a creatine-driven futile cycle, independent of UCP1, for thermogenesis. Specifically, TNAP dephosphorylates phosphocreatine to creatine, and the continual ATP-dependent rephosphorylation of creatine dissipates energy as heat [143–148]. The potential for exercise-induced beige adipocyte activation remains an area of ongoing research, particularly in the context of metabolic health and cold-environment adaptation.

### Adaptation of Adipose Tissue to Exercise Training

After the description of the different types of adipose tissues and adipocytes, it now makes sense to discuss their adaptation to exercise training. Like skeletal muscle, adipose tissue responds to chronic exercise. Transcriptomic studies have shown that adipose tissue responds to exercise training by a reduction of gene transcripts related to lipid uptake, synthesis and storage while also introducing a healthier circadian rhythm in adipose tissue from obese participants [149]. A healthier circadian rhythm is characterized by a stable, well-trained 24-h oscillation of behavioral, hormonal and metabolic processes that are aligned with the external light-dark cycle, thereby supporting optimal sleep, metabolic regulation and physiological function. Human WAT already responds to 3 weeks of moderate-intensity aerobic training by increasing its lipolytic capacity in obese participants [150]. This is likely due to the increase of circulating catecholamines (adrenaline and noradrenaline) in response to exercise, which activate β-adrenergic receptors in adipocytes and stimulate HSL-mediated lipolysis, thereby increasing the release of free fatty acids during exercise [151, 152]. Similarly, noradrenaline also activates cold-induced thermogenesis in human brown adipocytes via the β2-adrenergic receptor [24]. Collectively, these studies show that to understand the beneficial effects of different exercise intensities, we need to consider both the metabolic and the molecular response. The following paragraphs sum up how both WAT and thermogenic adipose tissue (beige adipocytes and BAT) respond to exercise. In addition, the long-term effect of skeletal muscle hypertrophy on the regulation of fat mass is discussed.

### Adaptation of White Adipose Tissue to Exercise Training

**Fig. 4:**
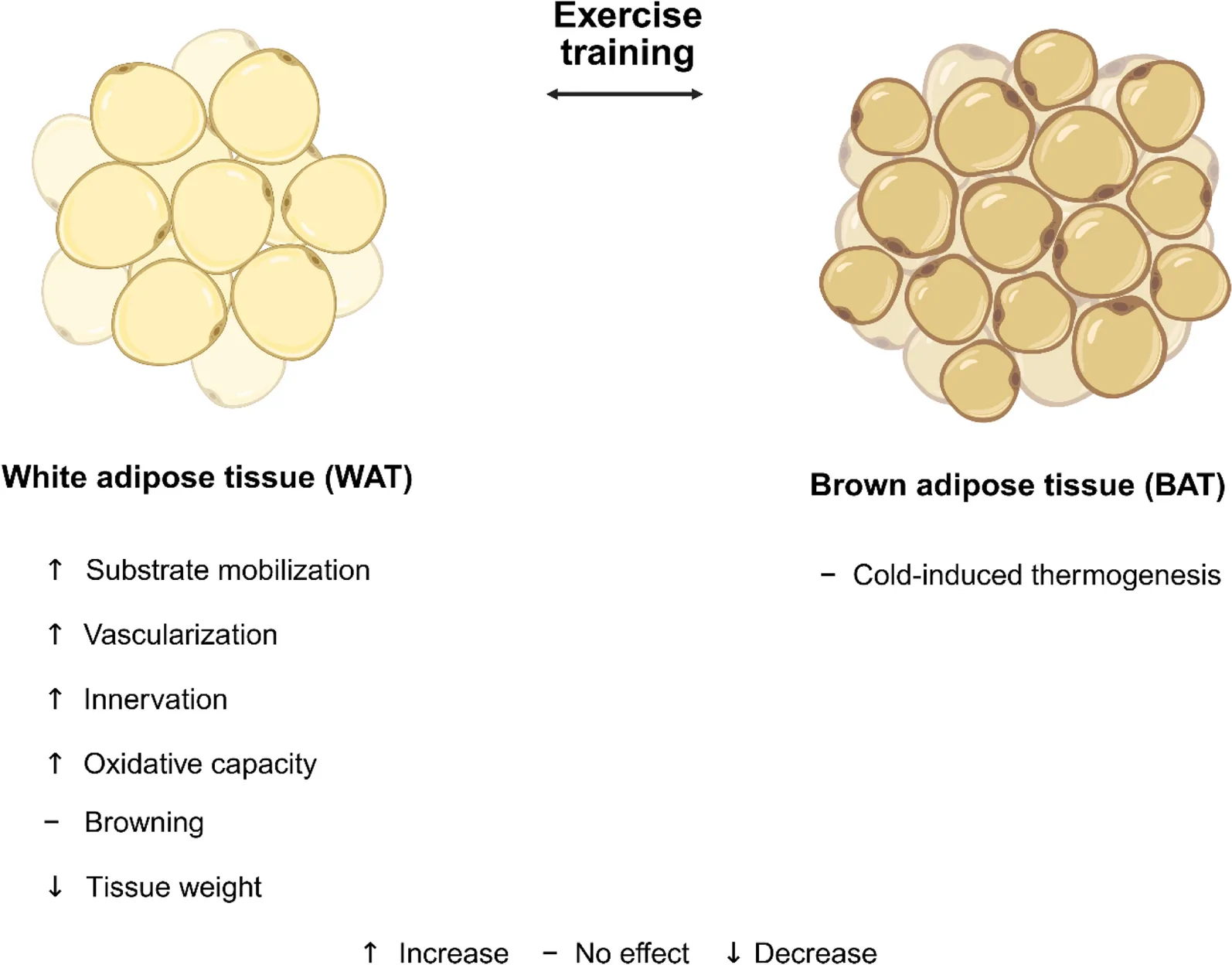
Schematic overview of exercise-induced adaptations in human white adipose tissue (WAT) and brown adipose tissue (BAT). Arrows indicate the direction of reported changes following chronic exercise training. Evidence for browning of WAT in humans remains limited. Original figure created by the authors based on previously published human studies

The Molecular Transducers of Physical Activity Consortium (MoTrPac) study demonstrated that exercise training induces systemic adaptations extending beyond skeletal muscle to virtually all organs, including adipose tissue [153]. Chronic exercise leads to a marked morphological and cellular remodeling of WAT [154–156] and these adaptations are summarized in the following sections. Figure 4 summarizes the exercise-induced adaptations on WAT and BAT among humans.

In human exercise intervention studies, subcutaneous WAT biopsies are typically obtained before and after exercise training. Using this approach, proteomic analyses of subcutaneous WAT collected after 8 weeks of high-intensity interval training (HIIT; three sessions per week involving rowing and cycling) revealed distinct protein expression profiles in lean men compared to men with type 2 diabetes mellitus [154]. Despite the relatively short intervention period, HIIT induced significant changes in proteins related to iron homeostasis, including ferritin light chain (FTL), ferritin heavy-chain-1 (FTH1) and reduced form of nicotinamide adenine dinucleotide (NADH) dehydrogenase (ubiquinone) 1 alpha subcomplex subunit 10 (NDUFA10) [154]. Increases in FTL and FTH1 expression within WAT were accompanied by reduced circulating serum ferritin concentrations and were associated with improvements in insulin sensitivity. These findings suggest that HIIT enhances the iron storage capacity of WAT, thereby lowering systemic iron availability and contributing to improved insulin homeostasis [154] (Fig. 4).

Exercise-induced remodeling of WAT structure and cellular composition has been further characterized in rodent models. In mice, 11 days of wheel running remodeled inguinal WAT by reducing collagen deposition while promoting vascularization and neuronal development [155]. Extending these observations in humans, 12 weeks of treadmill running performed three times per week increased the expression of neuronal growth factor 1 (NEGR1) in abdominal and gluteal subcutaneous WAT in obese women. NEGR1 may contribute to exercise-induced improvements in adipose tissue innervation and metabolic health [155] (Fig. 4).

Comprehensive analyses in mice further reveal that exercise induces depot-specific adaptations across subcutaneous WAT, visceral WAT and BAT, as evidenced by changes in gene and protein expression as well as metabolic function [156]. Within subcutaneous WAT, distinct depots respond differently to exercise training. Inguinal WAT from the groin area exhibits robust increases in genes associated with mitochondrial function, WAT browning, glucose metabolism and lipid oxidation, whereas subcutaneous WAT from the armpit area shows more limited transcriptional changes, primarily involving fatty acid oxidation [156]. Among the visceral depots, perirenal, mesenteric and perigonadal WAT were most responsive to exercise training. The perigonadal WAT displays the greatest responsiveness to exercise. Notably, perigonadal WAT demonstrates increased expression of genes related to mitochondrial activity, browning, glucose metabolism and lipid oxidation, whereas perirenal WAT predominantly shows downregulation of these pathways [156].

Collectively, these findings highlight pronounced adipose depot-specific adaptations to exercise training. Such heterogeneity may partly reflect the anatomical proximity of individual fat depots to metabolically active organs, particularly skeletal muscle. Potential mechanisms by which contracting muscle influences nearby adipose tissue, such as the release of exercise-induced signaling molecules, are discussed in detail in the Inter-organ Crosstalk During Exercise from and to Adipose Tissue section.

### Can Exercise Training Cause a Browning of White Adipose Tissue or Adaptations of Brown Adipose Tissue?

Another question is whether exercise training can promote white adipose tissue browning. The origin of this idea stems from a publication in Nature in 2012, that reported that exercise causes skeletal muscle to release a myokine termed “irisin” (encoded by the gene *FNDC5*) that stimulates UCP1 expression in beige and white adipocytes [26]. In the same year, another article reported no evidence for such a role in humans [157]. The effect of endurance training on adipose tissue thermogenesis and WAT browning has since been investigated in mice and humans.

In mice housed below thermoneutrality, endurance exercise consistently induces WAT browning, characterized by increased mitochondrial content, UCP1 expression, and oxidative capacity. However, when mice are housed at thermoneutrality, many of these browning responses to exercise are markedly attenuated or absent, indicating that cold stress strongly contributes to the apparent exercise-induced browning phenotype [158]. Furthermore, another study highlighted that commonly used murine exercise models may yield different metabolic and adipose tissue adaptations depending on housing temperature, which can substantially influence outcomes related to insulin action, energy expenditure and tissue remodeling. These findings underscore the importance of considering physiological context when translating exercise-induced changes in adipose tissue from animal models to humans, as ambient stressors may confound mechanisms that are assumed to be attributed to exercise training itself [159].

Human studies suggest that endurance or resistance exercise does not cause WAT browning as judged by, e.g., the expression of thermogenic gene markers in subcutaneous WAT [149, 160–163]. Similarly, positron emission tomography (PET) imaging studies have also failed to detect increases in BAT volume or activity following combined endurance and resistance exercise training for 24 weeks [138] (Fig. 4). The lack of WAT browning after exercise training is surprising because WAT browning factors such as noradrenaline or inosine increase in blood after exercise [137, 164] and cause WAT browning in cultured primary human brown adipocytes [165, 166]. However, acute exercise not only affects noradrenaline and inosine, but a plethora of other factors and the overall effect seems to be - no WAT browning. Thus, current evidence suggests that exercise training-induced browning of WAT in humans is a myth.

### Effect of Resistance Training and Muscle Hypertrophy on Adipose Tissue

Skeletal muscle and fat mass are inversely related, as the stimulation of global skeletal muscle hypertrophy typically leads to fat loss while muscle atrophy has opposite effects [167]. This phenomenon is known as “repartitioning” in the meat industry where drugs such as β2-agonists are used to increase muscle mass or meat while reducing fat mass [168]. Importantly, this is probably not because the muscle hypertrophy factor also acts on adipose tissue. For example, skeletal muscle-specific expression of Akt1 not only induces fast muscle hypertrophy, but is also sufficient to reduce fat depots by ~50% as a secondary effect in mice on a high-fat, high-sugar diet [169]. Similarly, for fat loss to occur, myostatin must be lost in skeletal muscle, whereas loss of myostatin signaling in adipose tissue does not reduce fat mass [170].

The mechanism by which the stimulation of muscle hypertrophy reduces fat mass is still unclear. In a recent review we have suggested two possible mechanisms [167]. The first is that hypertrophying muscles “steal” metabolites from adipose tissue and other organs and the second is that skeletal muscle releases a myokine that suppresses fat mass [170]. Whether such a myokine exists is currently unknown. In humans, skeletal muscle constitutes 20–50% of total bodyweight and is therefore the largest organ by volume [171]. Hypertrophying muscles are a “metabolite sink”, as they must take up one gram of metabolites from the circulation for every gram of dry biomass that they build [172]. Thus, if a person gains 1 kg of muscle mass (≈300 g of dry mass), then this means that 300 g of metabolites must be removed from the circulation and are unavailable to other organs such as adipose tissue. Hypertrophying muscles are also likely to take up risk factors for disease. The most obvious example is branched-chain amino acids (BCAAs) [173, 174] that are required for protein synthesis and that are a possible causal risk factor for developing diabetes [175], cardiovascular disease [174] and pancreatic cancer [176].

### Muscle Mass Preservation During Fat and Weight Loss

Caloric restriction not only leads to fat loss but also to a loss of lean mass that includes skeletal muscle [177]. For example, in the original semaglutide weight loss trial participants lost on average 9.2 kg (5.7–12.7 kg) fat mass and 5.4 kg (3.8–7.1 kg) lean mass [178]. This has triggered a scientific debate on how to prevent this loss of muscle mass. The problem is similar for bodybuilders in the cutting phase before a competition, as they must reduce fat mass to a minimum while preventing a loss of skeletal muscle mass. We now summarize evidence-informed training guidelines [179] to maintain skeletal muscle mass while losing fat mass.

Maintaining muscle mass during fat loss requires adherence to evidence-based diet and exercise protocols. Bodybuilding athletes, for instance, achieve single-digit body fat percentages through prolonged, controlled dieting (28 ± 8 weeks), targeting a gradual weight loss rate of 0.5 ± 0.2% of bodyweight per week [180]. This approach preserves energy availability for resistance training, which provides the stimulus for muscle retention. Recommended daily caloric intake ranges from 36 ± 5 kcal/kg body weight for men and 32 ± 3 kcal/kg body weight for women, respectively. Dietary fat intake is restricted to 0.6–0.8 g/kg body weight per day to allocate sufficient calories for protein and carbohydrate intake [180]. Current recommendations for muscle maintenance are a daily protein intake of 1.2–1.6 g/kg body weight, with high-quality protein sources (e.g., leucine-rich sources) consumed evenly across meals (≈30 g per serving) [181–183]. Besides sufficient protein intake, habitual resistance exercise remains a cornerstone of muscle preservation during fat loss diets, as the mechanical loading itself stimulates muscle protein synthesis and improves net protein balance for up to 48 h after a training session [184].

### Inter-organ Crosstalk During Exercise from and to Adipose Tissue

**Fig. 5:**
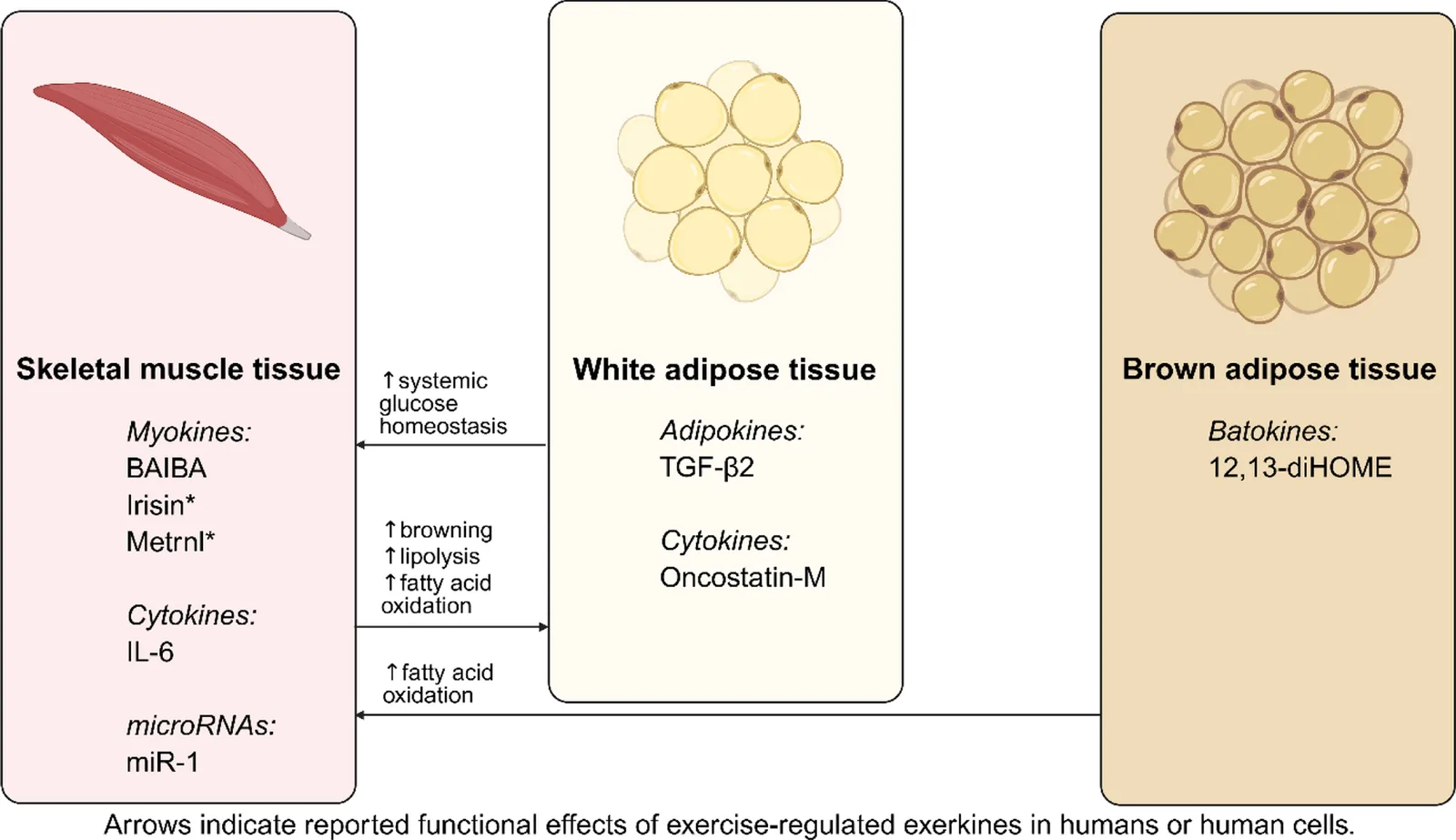
Exercise-regulated secreted factors (“exerkines”) with evidence from human studies. Schematic overview of inter-organ crosstalk between skeletal muscle and adipose tissues mediated by exercise-regulated secreted factors (“exerkines”) with evidence from human studies or human cell models. Arrows indicate reported functional effects on target tissues and do not imply that each listed factor mediates all effects. *For irisin and meteorin-like (Metrnl), evidence in humans is currently limited to circulating measurements and in vitro responses in human cells. *BAIBA* β-aminoisobutyric Acid, *Metrnl *meteorin-like, *IL-6* interleukin 6, *miR-1 *microRNA1, *TGF-β2 *transforming growth factor beta 2, *12,13-diHOME *12,13-dihydroxy-9Z-octadecenoic acid. Original figure created by the authors based on previously published human studies

Adipose tissue functions not only as an energy storage site but also as an endocrine organ, as it secretes hormone-like signaling molecules that mediate inter-organ communication during rest and exercise [185–187]. These factors are termed adipokines (e.g., adiponectin and leptin) if they are secreted from WAT or batokines (e.g., fibroblast growth factor 21) if they are secreted from BAT. Hormone-like signaling molecules secreted during exercise are additionally termed exerkines [188]. In the following sections, we summarize exerkines that have been validated in humans or, at a minimum, in human cell lines. Figure 5 illustrates the crosstalk between skeletal muscle, WAT and BAT in humans.

### Exerkines Released from Adipose Tissue

Both WAT and BAT secrete exerkines that induce endocrine, paracrine and/or autocrine effects in other organs, including skeletal muscle [188]. As outlined previously, WAT undergoes pronounced adaptations in response to exercise training. However, WAT also actively contributes to inter-organ communication through the release of adipokines. In mice, subcutaneous WAT increases circulating concentrations of transforming growth factor-β2 (TGF-β2) following 11 days of voluntary wheel running. Similarly, in humans, exercise training elevates TGF-β2 gene expression in subcutaneous WAT and increases circulating TGF-β2 concentrations. This rise in TGF-β2 has been shown to enhance glucose uptake in skeletal muscle cells and adipocytes derived from mice in vitro. Moreover, TGF-β2 attenuates inflammation and increases fatty acid oxidation as well as liver health in mice fed a high-fat diet [189] (Fig. 5).

Further evidence also points to oncostatin-M, a cytokine upregulated in WAT after acute exercise, as a potential mediator of crosstalk between adipose tissue and skeletal muscle. Transcriptomic profiling of human subcutaneous adipose tissue following about of exercise identified oncostatin-M among the most robustly induced secreted factors, with its expression arising predominantly from immune cell fractions (monocytes and macrophages) within WAT and the corresponding oncostatin-M receptor is present on white adipocytes [190]. In vitro experiments further show that oncostatin-M enhances mitogen-activated protein kinase (MAPK) signaling and increases lipolysis in human adipocytes, suggesting a role for this cytokine in coordinating metabolic and inflammatory adaptations of adipose tissue in response to exercise [190] (Fig. 5).

BAT, whose primary function is thermogenesis in response to cold exposure, also contributes to the regulation of skeletal muscle function through the secretion of batokines. For instance, BAT-derived myostatin has been shown to inhibit muscle growth [191]. In addition, the lipid-derived batokine 12,13-dihydroxy-9Z-octadecenoic acid (12,13-diHOME) is released from BAT during moderate-intensity exercise. Increased circulating levels of 12,13-diHOME enhance fatty acid uptake and oxidation in skeletal muscle, thereby supporting energy metabolism during exercise [192] (Fig. 5).

### Exerkines That Act on Adipose Tissue

As discussed previously, endurance exercise induces the release of several myokines from skeletal muscle that exert effects on adipose tissue. One prominent example is irisin, which has been linked to the induction of WAT browning in mice [26]. However, evidence supporting a comparable effect in humans remains limited. Similarly, the exercise-induced myokine meteorin-like (Metrnl) promotes thermogenic and anti-inflammatory gene programs in epididymal WAT in mice [193] (Fig. 5). For both irisin and Metrnl, current evidence in humans is largely restricted to in vivo measurements of circulating concentrations and in vitro responses of human adipocytes.

Forced expression of PGC1α in myocytes induces the secretion of β-aminoisobutyric acid (BAIBA), a small metabolite that promotes WAT browning and improves glucose homeostasis in mice. BAIBA has also been shown to induce browning-related gene expression in human pluripotent stem cells differentiated into adipocytes. In humans, circulating BAIBA concentrations are inversely associated with metabolic risk factors, suggesting a potential role in the modulation of metabolic health [194] (Fig. 5).

During endurance exercise, the cytokine IL-6 is released from skeletal muscle [195, 196]. IL-6 stimulates hepatic glucose output and promotes lipolysis in WAT [197]. Importantly, IL-6 appears to be required for exercise-induced reductions in visceral WAT, as antibody-mediated blockade of IL-6 in obese individuals abolishes the fat-reducing effects of exercise training [198]. Moreover, elevated IL-6 concentrations have been detected in subcutaneous abdominal WAT between 30 min and 3 h after cycling exercise [199], suggesting a role for IL-6 in mediating exercise-induced fat utilization and fatty acid oxidation (Fig. 5).

Resistance exercise is associated with reduced circulating myostatin concentrations in blood [200] and decreased myostatin expression in human skeletal muscle [201]. In mice, skeletal muscle-specific knockout of myostatin leads to pronounced muscle hypertrophy accompanied by reduced fat mass and improved glucose homeostasis [170, 200]. Although these findings do not indicate a direct action of myostatin on adipose tissue, they support the hypothesis that muscle hypertrophy induced by resistance exercise might release other myokines that explain how skeletal muscle regulates, e.g., the reduction of adipose tissue through resistance training [167].

In addition to soluble exerkines, inter-organ communication between skeletal muscle and adipose tissue is also mediated by extracellular vesicles (EVs), an increasingly recognized mechanism of exercise-induced signaling. EVs are membrane-derived vesicles that facilitate cell-to-cell communication and include exosomes (50–150 nm) and microvesicles (50–500 nm). These vesicles transport diverse molecular cargo, such as nucleic acids (including microRNA, non-coding RNA, mRNA, DNA, histones), proteins, and lipids. EV uptake by target cells occurs via mechanisms such as endocytosis, phagocytosis or direct membrane fusion, depending on vesicle type and cellular context [202]. EVs are released under both physiological and pathological conditions and have gained considerable attention as potential biomarkers and therapeutic targets [203, 204].

Endurance exercise has long been recognized as a modulator of circulating EVs' abundance and composition. Recent studies have leveraged exercise training interventions to identify novel exerkines packaged within EVs [205]. For example, a 12-week combined endurance and resistance training program in African women altered microRNA expression profiles in WAT, with specific microRNAs showing inverse associations with HDL-cholesterol concentrations, a key indicator of metabolic health [206]. Moreover, resistance exercise has been linked to increased expression of muscle-derived microRNA-1 (miR-1), which has been shown to increase lipolysis in adipose tissue [207] (Fig. 5).

Collectively, these findings indicate that exercise-induced communication between skeletal muscle and adipose tissue is mediated not only by circulating exerkines but also by EV-associated molecular cargo, thereby expanding the spectrum of inter-organ signaling mechanisms activated by exercise training.

## Conclusions

In summary, “fat” - as a molecule and tissue - is relevant in the context of exercise biology and training adaptations. Lipids not only serve as a major metabolic fuel during exercise, but also act as important signaling molecules that regulate cellular and systemic metabolism. The existence of white, brown, and beige adipocytes highlights the heterogeneity of adipose tissue and underscores its role as a metabolically active organ that communicates with other tissues, including skeletal muscle and the liver. Like other endocrine organs, adipose tissue secretes hormone-like factors into the circulation, which can either improve or impair metabolic health and influence the risk of cardiovascular disease. Exerkines released by adipose tissue seem to be understudied when compared to skeletal muscle research.

Despite decades of research, there are still major research questions in relation to exercise and adipose tissue:

1. Training: Which forms of exercise training and other interventions best enhance skeletal-muscle β-oxidation capacity and increase fat utilization during a given exercise intensity? What are the mechanisms of adaptation?
2. Training: What is the depot-specific adaptation of subcutaneous versus visceral white adipose tissue during exercise training, and how do these contribute to metabolic health, substrate availability, and performance?
3. Training: How do white, brown and beige adipocyte populations as well as other adipose tissue cells remodel in response to endurance versus resistance training versus pharmacological stimulation of muscle hypertrophy in humans?
4. Molecular regulation: What molecular signals from exercising muscle (myokines, metabolites, EV cargo, neural input) trigger adaptations in white and brown adipose tissue or vice versa?
5. Molecular regulation: Do exercise-released adipokines and batokines (e.g., adiponectin, leptin, FGF21, Nrg4) contribute to improvements in endurance performance, substrate utilization, recovery, or thermoregulation?
6. Performance: What is the role of BAT activation (cold exposure, pharmacology, nutrition, exercise training) in human athletic contexts?
7. Clinical: How do large fluctuations in body fat during cutting/bulking cycles, e.g., in bodybuilders or during on/off semaglutide usage, alter adipose tissue populations, adipose endocrine function, inflammation, and adipokine/batokine profiles?
8. Clinical: How does chronic low energy availability alter adipose-tissue function including lipolysis, adipokine secretion, and intracellular lipid metabolism and to what extent do these changes contribute to the metabolic, hormonal, and performance impairments observed in RED-S?

Importantly, given adipose differences between women and men, these questions should be researched using equal numbers of female and male participants.

## Data Availability

Not applicable.

## Abbreviations

ACSL: Acyl-CoA synthetase
acyl-CoA: Acyl-coenzyme A
AMPK: Adenosine monophosphate-activated protein kinase
ATGL: Adipose triglyceride lipase
ATP: Adenosine triphosphate
BAIBA: β-Aminoisobutyric acid
BAT: Brown adipose tissue
BCAAs: Branched-chain amino acids
BMI: Body mass index
cAMP: Cyclic adenosine monophosphate
CACT: Carnitine-acylcarnitine translocase
CPT1: Carnitine palmitoyltransferase 1
CREB: C-responsive element-binding protein
CT: Computer tomography
EVs: Extracellular vesicles
FABP: Fatty acid-binding protein
FAPs: Fibro-adipogenic progenitors
FAT/CD36: Fatty acid translocase
FATP: Fatty acid transport protein
Fatmax: Exercise intensity eliciting the individual maximum fat oxidation rate
FGF21: Fibroblast growth factor 21
FTH1: Ferritin heavy-chain-1
FTL: Ferritin light chain
FNDC5: Fibronectin type III domain-containing protein 5
HDL: High-density lipoprotein
HIIT: High-intensity interval training
HSL: Hormone sensitive lipase
IL-6: Interleukin-6
IMTG: Intramuscular triglyceride
LDL: Low-density lipoprotein
MAPK: Mitogen-activated protein kinase
Metrnl: Metformin-like
MFO: Maximum fat oxidation
MGL: Monoglyceride lipase
miR-1: MicroRNA-1
MoTrPAC: Molecular Transducers of Physical Activity Consortium
mTOR: Mammalian target of rapamycin
NADH: Nicotinamide adenine dinucleotide dehydrogenase
NDUFA10: NADH ubiquinone 1 alpha subcomplex subunit 10
NEGR1: Neuronal growth factor 1
Nrg4: Neuregulin 4
NSAID: Nonsteroidal anti-inflammatory drug
P: Phosphate
PET: Positron emission tomography
PGC-1α: Peroxisome proliferator-activated receptor, gamma coactivator 1, alpha
RED-S: Relative energy deficiency in sport
RER: Respiratory exchange ratio
scRNAseq: Single-cell RNA sequencing
S1P: Sphingosine 1 phosphate
TCA cycle: Tricarboxylic acid cycle
TGF-β2: Transforming growth factor beta 2
TNAP: Tissue non-specific alkaline phosphatase
TNFα: Tumor Necrosis Factor alpha
UCP1: Uncoupling protein 1
V̇CO2: Carbon dioxide production
V̇O2: Oxygen uptake
WAT: White adipose tissue
Wmax: Maximal power output
12,13-diHOME: 12,13-Diydroxy-9Z-octadecenoic acid
12-HEPE: 12-Hydroxyeicosapentaenoic acid
18FDG: 18 Fluorodeoxyglucose
